# Role of Sam68 as an adaptor protein in inflammatory signaling

**DOI:** 10.1007/s00018-023-05108-9

**Published:** 2024-02-14

**Authors:** Vemana Gowd, Joseph D’Amato Kass, Nandini Sarkar, Parameswaran Ramakrishnan

**Affiliations:** 1 Department of Pathology, School of Medicine, Case Western Reserve University and University Hospitals Cleveland Medical Center, 6526, Wolstein Research Building, 2103 Cornell Road, Cleveland, OH 44106 USA; 2 The Case Comprehensive Cancer Center, School of Medicine, Case Western Reserve University, Cleveland, OH 44106 USA; 3 Department of Biochemistry, School of Medicine, Case Western Reserve University, Cleveland, OH 44106 USA

**Keywords:** Sam68, Inflammation, Inflammatory bowel disease, Arthritis, Cardiovascular disease, TNF, TLR, TCR, BCR

## Abstract

Sam68 is a ubiquitously expressed KH-domain containing RNA-binding protein highly studied for its involvement in regulating multiple steps of RNA metabolism. Sam68 also contains multiple protein–protein interaction regions such as proline-rich regions, tyrosine phosphorylation sites, and arginine methylation sites, all of which facilitate its participation as an adaptor protein in multiple signaling pathways, likely independent of its RNA-binding role. This review focuses on providing a comprehensive report on the adaptor roles of Sam68 in inflammatory signaling and inflammatory diseases. The insights presented here have the potential to open new avenues in inflammation research and justify targeting Sam68 to control aberrant inflammatory responses.

## Introduction

**Fig. 1:**
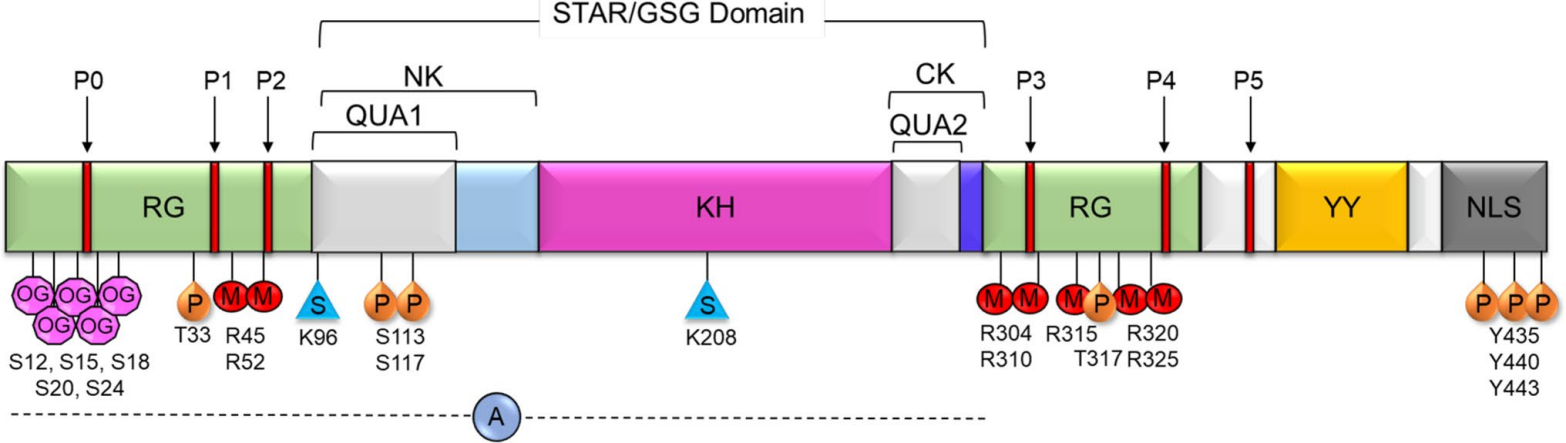
Schematic representation of Sam68 domains and posttranslational modifications. P0–P5—proline-rich regions, RG—arginine/glycine-rich regions, QUA1 and QUA2—domains flanking KH domain where QUA1 mediates dimerization of Sam68, KH—hnRNP K homology domain, NK—N terminal of KH, CK—C terminal of KH, YY—tyrosine phosphorylation sites, NLS—nuclear localization signal. Known posttranslational modifications of Sam68 are annotated at the respective residues. OG—O-GlcNAcylation, P—phosphorylation, M—methylation, S—SUMOylation, A—acetylation region

Src-Associated in Mitosis 68 kDa Protein (Sam68), also known as KH Domain-Containing, RNA-Binding, Signal Transduction-Associated Protein 1 (KHDRBS1), belongs to the signal transduction and activation of RNA (STAR) family of proteins [1]. Sam68 plays key roles in cell proliferation, differentiation, development, signal transduction, and various aspects of RNA metabolism such as transcription, alternative splicing, and nuclear export [2, 3]. Sam68 contains a heteronuclear ribonucleoprotein particle K (hnRNP K) homology (KH) domain like other RNA-binding proteins in the STAR family. The KH domain is critical for the recognition and subsequent binding of Sam68 to the 3′ U(C/A)A(C/U) moiety commonly found on RNA. The KH domain is flanked by the highly conserved QUA1 region within the N-terminus (NK) and QUA2 region within the C-terminus (CK) of KH domain [3] (Fig. 1). The QUA1 region plays an important role in Sam68 dimerization, while the QUA2 region is responsible for recognizing a specific RNA sequence and stabilizing the dimer orientation via contact with the QUA1 and KH regions [3]. Together, the NK/QUA1, KH, and CK/QUA2 regions make up the STAR domain or GRP33/SAM68/GLD-1 (GSG) domain that is essential for RNA binding and homodimerization [1]. In addition to the STAR/GSG domain, Sam68 contains various regions that mediate protein–protein interactions as well as multiple posttranslational modification sites (Table 1). The C-terminus of Sam68 contains tyrosine residues which are phosphorylated, which mediate binding to SH2 domain-containing proteins (Table 1) [2]. The protein’s six proline-rich regions allow for binding to SH3 domains of Src kinases, which regulate a wide variety of cellular functions such as cell proliferation, survival, and migration [4–6]. The tyrosine phosphorylation-dependent SH2 domain protein binding and proline-rich region-dependent SH3 domain binding have been shown to inhibit Sam68’s interaction with RNA and facilitate its function as an adaptor molecule in signaling involved in cell cycle progression and cell proliferation (Table 1) [2]. In addition to phosphorylation, other posttranslational modifications, including O-GlcNAcylation [7], arginine methylation [8], SUMOylation [9], and acetylation [10], control cellular functions of Sam68 (Table 1). Lin et al., reported that the O-GlcNAcylation of Sam68 plays a role in modulating lung cancer cell migration and invasion [7]. Arginine methylation of Sam68 by the protein arginine methyltransferase 1 (PRMT1) has been shown to decrease the RNA-binding ability of Sam68, modulating its protein interactions and controlling its localization and function [8, 11]. Sam68 is SUMOylated by SUMO ligase PIAS1, which results in the repression of cyclin D1 leading to G1 arrest and inhibition of cell proliferation (Table 1). However, it is unclear whether SUMOylation of Sam68 has any effect on the RNA-binding affinity of Sam68 and protein–protein interactions [9]. The RNA-binding domain is required for Sam68 apoptosis induction, while acetylation of Sam68 enhances its RNA-binding ability and may upregulate pro-apoptotic proteins [9]. Sam68 also contains a nuclear localization signal (NLS) in its C-terminus which allows the protein to move between the cytoplasm and nucleus of the cell, as well as execute its many transcriptional and posttranscriptional regulatory roles [2].

**Table 1:** Posttranslational modifications of Sam68 and their biological significance

Posttranslational modifications	Domains	Biological function
Phosphorylation	RG, NK, NLS	Decreases RNA-binding affinity of Sam68, promotes cell cycle progression, and cell proliferation [2]
Methylation	RG	Decreases RNA-binding affinity of Sam68, and upregulates Sam68–protein interactions [8]
SUMOylation	RG, KH	Promotes cyclin D1 repression leading to G1 arrest and decreases cell proliferation [9]
O-GlcNAcylation	RG (N-terminus)	Increases cell migration and invasion [7]
Acetylation	RG, NK, KH, CK	Increases RNA binding affinity of Sam68 and apoptosis [10]

*RG* Arginine/glycine-rich regions, *NK* N-terminal of KH, contains QUA1 region, *NLS* Nuclear localization signal, *KH* Heterogeneous nuclear ribonucleoprotein particle K (hnRNP K) homology domain, *CK* C-terminal of KH, contains QUA2 region

The lion’s share of the studies on Sam68 focuses on its roles in RNA binding and cancer [1, 12, 13]. Sam68 has also been shown to play a critical role as an adaptor protein in various signaling pathways such as insulin [14, 15], leptin [14, 15], T-cell receptor (TCR) and B-cell receptor (BCR) [14, 16], tumor necrosis factor (TNF) [6], and toll-like receptor (TLR) [17]. Recent findings suggest that higher expression levels of Sam68 are not only associated with various human cancers [1, 2, 18], but also with inflammatory diseases such as inflammatory bowel disease [19], arthritis [20], and arterial injury [21]. For example, the high levels of Sam68 observed in patients with ulcerative colitis (UC) correlated with higher levels of apoptosis and NF-κB activation [19]. As such, Sam68’s role in driving inflammation through NF-κB signaling and inhibition of wound repair processes has been linked to worsened patient outcomes across various inflammatory diseases [19, 21]. Understanding Sam68-dependent inflammatory signaling pathways and delineating the multifaceted effects of Sam68 on inflammation may improve patient prognosis and drive therapeutic development for inflammatory diseases. In this review, we highlight Sam68’s adaptor role in inflammatory diseases and discuss its potential as a therapeutic target.

### Sam68 as an adaptor protein

Adaptor proteins are essential for the propagation, amplification, and regulation of cellular signals [22]. In inflammatory signaling, adaptor proteins may play an essential role in both the specificity and diversity of cellular responses to inflammation, as well as to resolve inflammatory signaling before it becomes pathologic [23]. Sam68, first identified as an RNA-binding protein, has been shown to serve as an adaptor protein in an array of inflammatory signaling events, notably at the upstream-receptor level.

### Role of Sam68 in TCR signaling

**Fig. 2:**
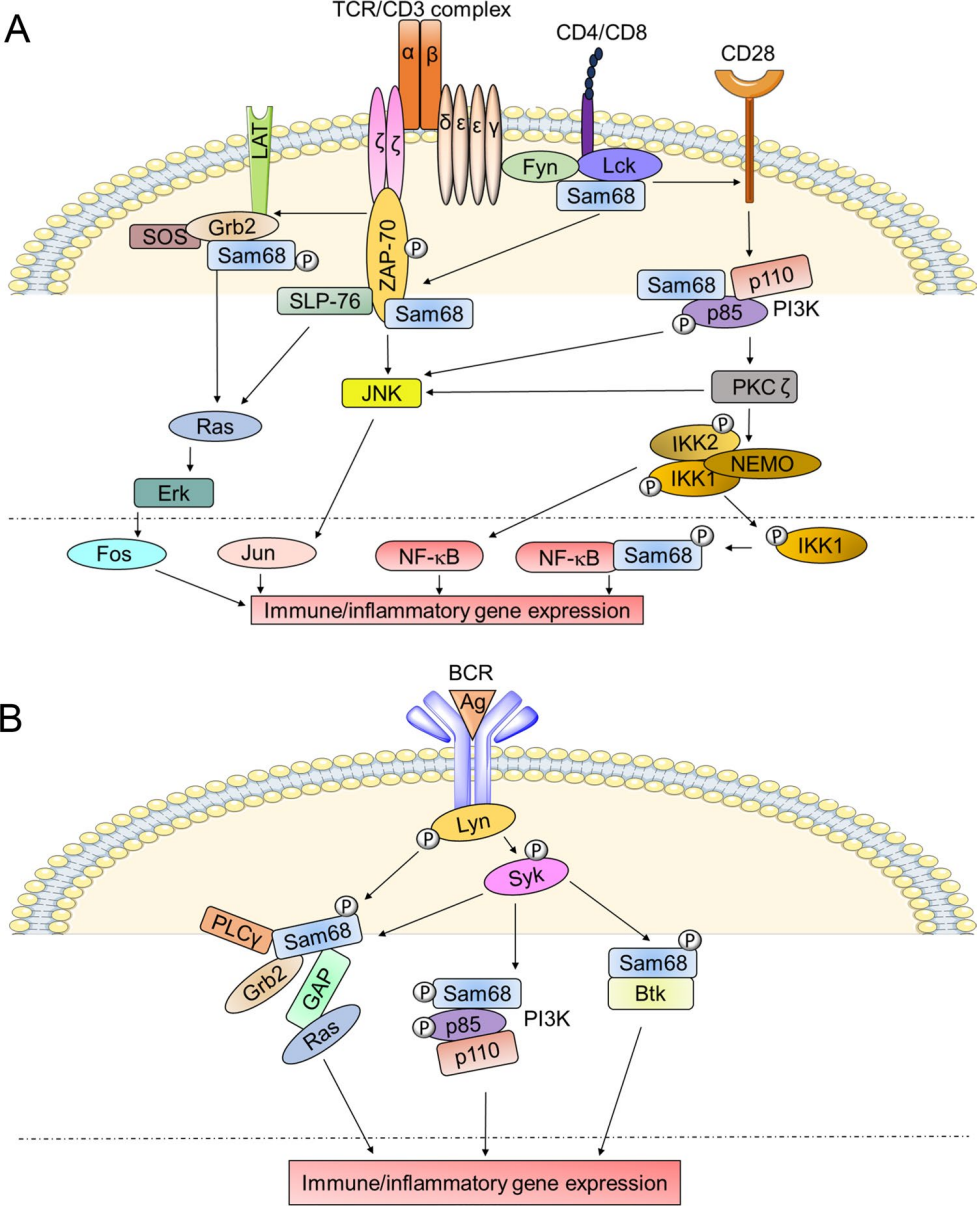
**A** Schematic representation of the role of Sam68 in TCR signaling. Upon TCR activation, multiple tyrosine residues are phosphorylated at immunoreceptor tyrosine-based activation motifs (ITAM) of the TCR–CD3 complex by the Src-family protein-tyrosine kinase LCK. Both LCK and Fyn contain SH3 and SH2 domains which may bind to naïve and phosphorylated Sam68, respectively. This is followed by the recruitment and activation of ZAP-70 and the scaffolding protein, SLP-76, which may bind to phosphorylated Sam68 through their SH2 domains. Signaling through SLP-76 and Grb2, which contain both SH2 and SH3 domains to facilitate Sam68 binding, will lead to activation of the MAP kinase pathway. The engagement of CD28 and co-stimulation of the TCR will lead to the activation of PI3 kinase and PKC, resulting in MAP kinase and IKK–NF-κB pathway activation. Activated IKK1 has also been shown to phosphorylate Sam68 in the nucleus and promote its binding to NF-κB p65 and activate CD25 expression. **B** Schematic representation of the role of Sam68 in BCR signaling. Upon BCR activation, through antigen binding, kinases such as Lyn and Btk, which contain both SH3 and SH2 domains, are recruited to the receptor complex. Sam68 may bind to the receptor complex through a proline-rich region—SH3 interaction in its naïve state, and when phosphorylated, Sam68 may bind to other proteins such as Syk through SH2 domain interactions. Downstream signaling proteins such as Grb2, GAP, and PLCγ contain both SH2 and SH3 domains which may facilitate binding to Sam68 and lead to Ras activation. Phosphorylated Sam68 will also interact with the PI3K’s SH2 domain to control downstream signaling events in B cell receptor signaling

The TCR enables T cells to recognize foreign antigens and signal to mount immune and inflammatory responses. TCR activation initiates a signaling cascade which triggers the recruitment of several cytoplasmic proteins to the receptor [24, 25]. The presence of proline-rich regions allows for Sam68’s interactions with SH3 domain containing adaptors such as Grb2 (Fig. 2A) [26] and the Src family tyrosine kinases, Fyn and Lck [27, 28]. These interactions may occur at the basal state, as they do not involve any stimulus-dependent posttranslational modifications. The six proline-rich domains throughout Sam68 are notably not clustered to one particular terminus, resulting in a spatially heterogeneous distribution of SH3 domain-binding motifs on Sam68 [29]. It is also possible that the conformational changes in the three-dimensional structure of Sam68, upon carboxy-terminal SH2 binding, may allow for a differential display of one or more proline-rich regions. Importantly, this would promote more efficient binding to SH3 domain containing proteins or binding to more than one SH3 domain containing protein, facilitating an essential adaptor/scaffolding role in propagating downstream TCR signaling. Furthermore, the binding of one SH3 domain-containing protein may also expose additional proline-rich regions or result in tyrosine phosphorylation of Sam68, thus allowing additional recruitment of both SH3 and SH2 domain containing proteins.

In contrast, Sam68’s binding to SH2 domain containing proteins is dependent on its tyrosine phosphorylation, which may happen only after TCR activation. TCR engagement results in the recruitment of several proteins along with multiple kinases such as Lck and Fyn, both of which contain SH3 and SH2 domains to facilitate the binding of non-phosphorylated and phosphorylated forms of Sam68. It is possible that the initial binding of Sam68 to the TCR complex is mediated through its interaction with the SH3 domain-containing proteins followed by its tyrosine phosphorylation. It is observed in in vitro studies that once tyrosine phosphorylation has occurred, Sam68 may also bind to the adaptor proteins such as ZAP-70 and SLP-76 and phosphoinositide 3-kinase (PI3K) as well as activate mitogen-activated protein kinase (MAPK) pathways (Fig. 2A) [25, 30]. Thus, through the interactions via proline-rich regions and tyrosine phosphorylated regions, Sam68 may perform its adaptor function in linking multiple proteins’ binding to the ζ chain of the TCR’s CD3, as well as to the intracellular domains of CD4/CD8, CD28, and linker for activation of T cells (LAT) (Fig. 2A).

Absence of Sam68 has been shown to significantly reduce the TCR-induced inflammatory interferon (IFN)-γ production in T helper 1 (Th1) cells. This was primarily attributed to Sam68’s ability to bind and inhibit microRNA-29, which inhibits the transcription factors T-bet and Eomes which control IFN-γ expression in T cells [31]. It remains to be investigated whether the adaptor function of Sam68 at the TCR also contributes to IFN-γ expression in T cells. Furthermore, Sam68 has been shown to be an essential non-Rel component of the NF-κB complex inducing CD25 expression in T cells [16] (Fig. 2A). T-cell activation results in IKKα-dependent serine phosphorylation of nuclear Sam68 and its binding to the NF-κB p65 subunit, and subsequent binding of this Sam68-p65 complex to the CD25 promoter. This alludes to an important nuclear-adaptor role of Sam68 in regulating gene expression. Because Sam68-p65 binding does not directly involve SH2/SH3 domain-dependent interactions, knowledge of the precise mechanism of Sam68-p65 binding in T cells is expected to reveal novel binding mechanisms through which Sam68 executes its adaptor role in the nucleus. Notably, some contradictions exist within the cytoplasmic adaptor role of Sam68 in TCR signaling. In a well-performed study, Fu et al. [16] showed that siRNA suppression of Sam68 in Jurkat cells did not prevent TCR activation and that PMA/ionomycin stimulation induced IκBα degradation. This raised the possibility that Sam68 may not be essential in cytoplasmic signaling events in T cells leading to NF-κB activation. However, the siRNA-mediated suppression of Sam68 in this study leaves a significant amount of residual Sam68 in the cells, which may suffice for signal transduction. Hence, to derive firm conclusions on the adaptor role of Sam68 in TCR signaling, it is necessary to conduct comprehensive studies using complete Sam68 knockout T cells and delineate downstream signaling following both CD3 activation alone and with CD3 and CD28 co-stimulation. Moreover, given the presence of multiple potential binding partners for Sam68 in the TCR complex and in the nucleus, a clearer understanding of the direct and indirect binding partners of Sam68 is necessary to elucidate the biochemical mechanisms by which Sam68 adapts TCR signaling.

### Role of Sam68 in BCR signaling

B cells are another major component of the adaptive immune system with a major role in antibody production [32]. They also partake in antigen presentation and secrete multiple cytokines that control immune response [33]. The BCR recognizes and binds to foreign antigens, leading to B cell activation and propagation of signaling via phosphorylation of multiple kinases including Lyn, Btk and PI3K at the BCR complex [34]. Sam68 interacts with the PI3K–SH2 domain through its phosphotyrosine region [34, 35] and the Btk–SH3 domain through its proline-rich regions (Fig. 2B) [36]. The proline-rich regions were also shown to control B cell signal transduction regulating cell growth and death [4]. Lyn kinase has been shown to phosphorylate Sam68 [37], but its direct interaction with Sam68 in B cells remains to be demonstrated. Lyn kinase has also been shown to be required for the activation of Syk kinase, which involves trans- and auto-phosphorylation events [38]. It is highly likely that the activated Syk kinase may interact with and phosphorylate Sam68 propagating downstream BCR signaling involving Lyn and Btk kinases [36, 38–40] based on the potential of Syk to phosphorylate Sam68 in vitro [41]. Sam68 has also been shown to interact with several other SH2 and SH3 domain-containing proteins that are associated in the BCR complex such as Grb2, PLC-gamma, and RAS GTPase activating protein (Ras-GAP) [42, 43]. Through this multiprotein binding ability, Sam68 is likely to control downstream signaling pathways activated following BCR stimulation such as NF-κB, nuclear factor of activated T-cells (NFAT), p38, JNK, ERK, and AKT. The role of Sam68 in adapting these kinases/pathways in BCR signaling remains a vast area inviting comprehensive investigation.

### Role of Sam68 in insulin receptor (IR) signaling

**Fig. 3:**
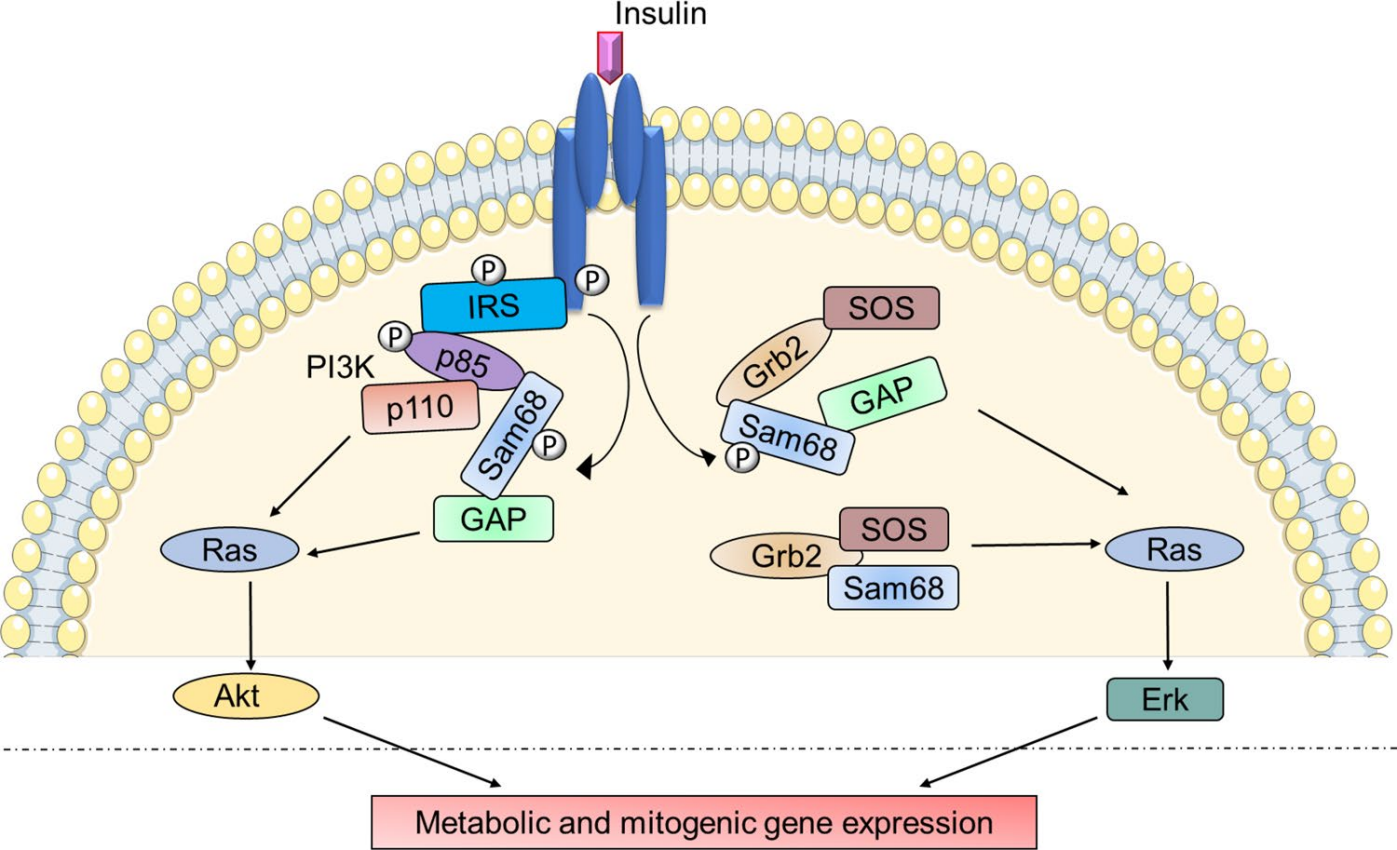
Schematic representation of the role of Sam68 in IR signaling. Insulin binding induces the phosphorylation of IR subunits and tyrosine phosphorylation of IRS1. Sam68 is tyrosine phosphorylated by the activated IR and the phosphorylated Sam68 associates with p85-PI3K via SH2 domains of the p85 subunit and recruits p120GAP to the PI3K pathway which links PI3K with Ras and AKT pathway downstream of IR signaling. Phosphorylated Sam68 interacts with the SH2 domains of GAPs promoting the association of GAP with the Grb2–SOS complex and downstream signaling to Ras/Erk pathway. Sam68 is also known to associate with the SH3 domains of Grb2 independent of insulin stimulation and activates Ras signaling

Insulin binding to the IR results in tyrosine phosphorylation of insulin receptor substrate 1 (IRS1) and downstream signaling cascade activation [44]. Sam68 has been shown to bind to IRS1 at the basal state and this interaction enhances following insulin stimulation, with an associated increase in Sam68 protein level both in the cytoplasm and at the IR. At the IR, Sam68 associates with PI3K via the SH2 domain of the p85 subunit (Fig. 3), a main downstream constituent of insulin signaling [46]. Insulin signaling also causes tyrosine phosphorylation of Sam68 enabling its interaction with SH2 domain containing proteins such as GAP and PI3K, both in vitro and in vivo [26, 35, 45]. Sam68 also has the ability to bind and recruit p120GAP to the PI3K pathway [26], possibly through interaction with the SH2 domain. The simultaneous association of Sam68 with p85 PI3K and GAP help to bring these two molecules together, thus linking PI3K with the RAS pathway in IR signaling (Fig. 3) [35, 47]. On the other hand, Sam68 is known to be associated with the SH3 domains of Grb2 independent of insulin stimulation in vivo; however, insulin stimulation has been shown to promote Sam68 phosphorylation and its association with the SH2 domains of GAP. This in turn promotes association of GAP with the Grb2–SOS complex. Both of these complexes lead to the downstream activation of the MAP kinase pathway, influencing metabolic and mitogenic gene expression (Fig. 3) [26]. Overall, current evidences show that Sam68 is a substrate phosphorylated in insulin signaling, and that it also acts as an adaptor linking multiple proteins at the IR. Most of the current knowledge on the role of Sam68 in IR signaling are derived from in vitro studies using cell lines and their physiological significance remains to be validated through in vivo studies. Possible IR-independent adaptor roles of Sam68 in the cytoplasm that may control inflammatory signaling and insulin resistance, as well the potential nuclear roles of Sam68 regulating insulin-dependent gene expression, are interesting areas pending investigation. The relationship between inflammation and insulin resistance has been well studied [48–50], but there remain further avenues of investigation. For example, pro-inflammatory cytokines are also known to induce the activation of serine and threonine kinases, which can in turn cause inhibitory phosphorylation of IRS1 and impede downstream signaling by IRS1 [44]. Whether inflammation in the context of IR signaling also causes serine/threonine phosphorylation of Sam68 has yet to be determined.

### Role of Sam68 in leptin receptor signaling

**Fig. 4:**
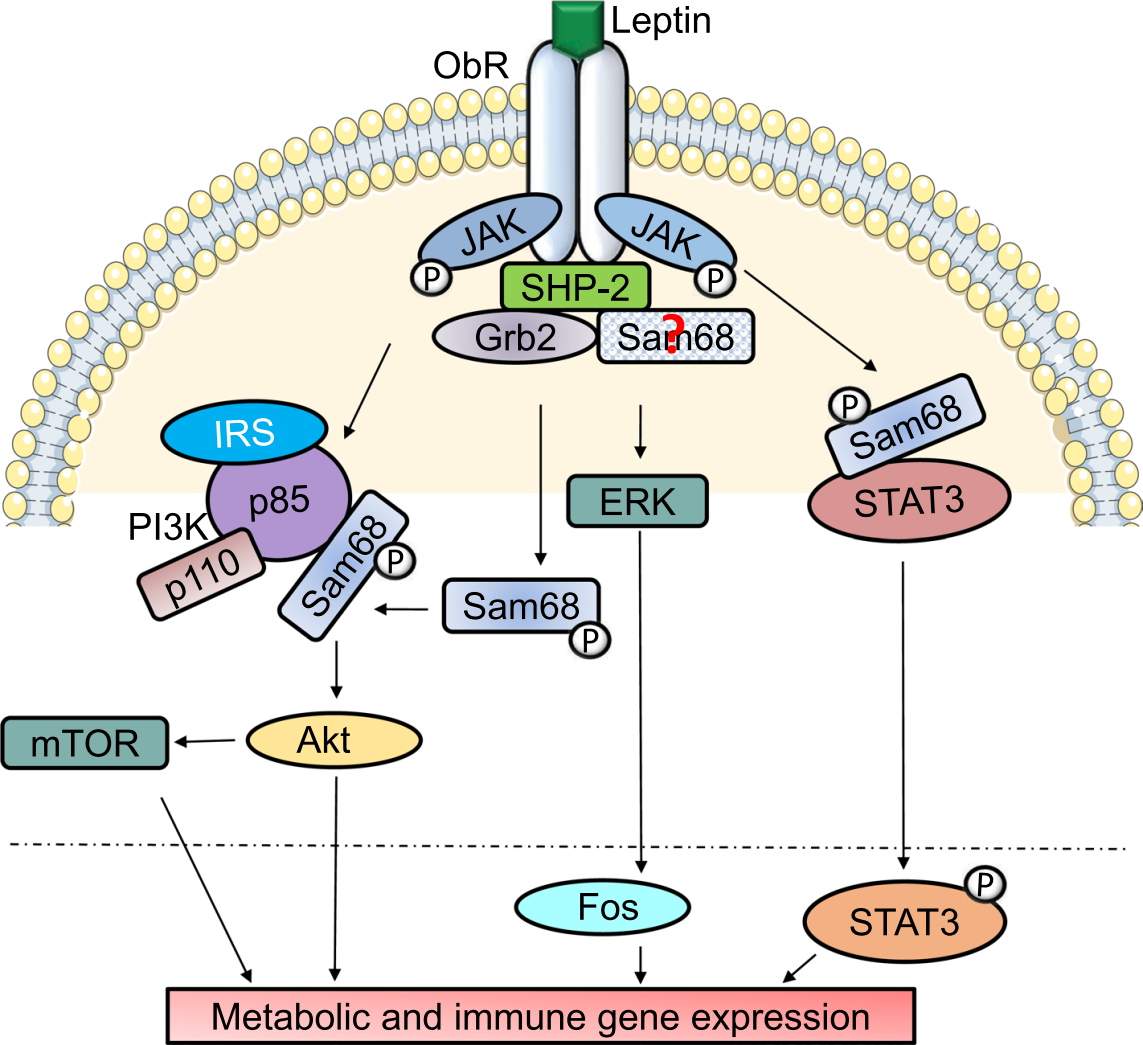
Schematic representation of the role of Sam68 in leptin receptor signaling. The binding of leptin to its receptor results in the formation of Ob-Rb/JAK complex that leads to cross-phosphorylation. This causes tyrosine phosphorylation of Sam68 which may promote Sam68’s interaction with SH3/SH2 domain containing protein such as Grb2. The binding of leptin to its receptor also results in the phosphorylation of SHP-2. Activated SHP-2 binds to phospho-tyrosine 985 of Ob-Rb which is critical for ERK activation by the leptin receptor, a pathway which might involve Sam68. Hypothetical association of Sam68 with SHP-2 is denoted with a question mark (?). OB-Rb activation causes ERK activation that leads to downstream Fos activation. Sam68 is tyrosine phosphorylated following OB-Rb activation and phosphorylated Sam68 may interact with PI3K’s SH2 domain and engage in downstream activation of Akt and mTOR pathways. Leptin also induces the association of phosphorylated Sam68 with STAT3 via SH2 domain interaction, which may result in STAT3 activation

Sam68 regulates leptin receptor signaling both through its RNA-binding function and as an adaptor protein. First, through its RNA-binding role, Sam68 has been shown to skew leptin receptor mRNA expression to its long isoform under physiological conditions [51]. The long isoform of the leptin receptor, or OB-Rb, facilitates pro-inflammatory responses in macrophages by inducing the secretion of the cytokines IL-6 and TNF [52, 53]. Importantly, the activation of pro-inflammatory receptors such as TLR4 (LPS activation), tumor necrosis factor receptor-1 (TNFR1) (TNF activation), and IL-1R (IL-1β activation) increases the circulating levels of leptin, which in turn is able to drive increased serum levels of the pro-inflammatory cytokines, e.g., TNF, IL-6, and IL-12 [53]. What then forms is a positive-feedback loop of inflammatory cytokine release, initially spawned by adipokines dependent upon Sam68 as an adaptor within the leptin receptor’s downstream signaling cascade. Activation of the leptin receptor triggers multiple pathways such as JAK–STAT, MAPK, and PI3K [54]. Signaling through OB-Rb induces Sam68 tyrosine phosphorylation resulting in decreased poly(U)-binding capacity, limiting mRNA processing function of Sam68 [51], and increasing its association with PI3K’s SH2 domain (Fig. 4). Similarly, leptin also stimulates tyrosine phosphorylation of Sam68 in a time and dose dependent manner, promoting its association with STAT3 via SH2 domain interaction in vitro (Fig. 4) [14]. To understand the molecular mechanisms of leptin receptor signal transduction pathway involving Sam68, it is necessary to first identify the SH2 domain containing proteins that are activated by the leptin receptor. SH2 domain containing protein tyrosine phosphatase 2 (SHP-2) is one such protein that has been shown to bind to phospho-tyrosine 985 of Ob-Rb upon leptin treatment; the bound SHP-2 itself was tyrosine phosphorylated following leptin stimulation (Fig. 4). While STAT3 downstream phosphorylation has been shown to be independent of SHP-2 phosphorylation, Li and Friedman have shown that the downstream-phosphorylation level of JAK2 is significantly decreased upon SHP-2 activation via leptin receptor signaling [55]. However, it is not yet known whether Sam68 has any role in SHP-2 regulated leptin receptor signal transduction through binding to the SH2 domain of SHP-2. It would prove interesting to see if Sam68 has a role in facilitating SHP-2 activation, which may then result in modulation of the leptin receptor signaling cascade. The current understanding on the role of Sam68 in leptin receptor signaling is mostly based on in vitro studies. Hence, further comprehensive in vivo studies are necessary to delineate the physiological role of Sam68 in leptin receptor signaling.

### Role of Sam68 in TNFR1 signaling

**Fig. 5:**
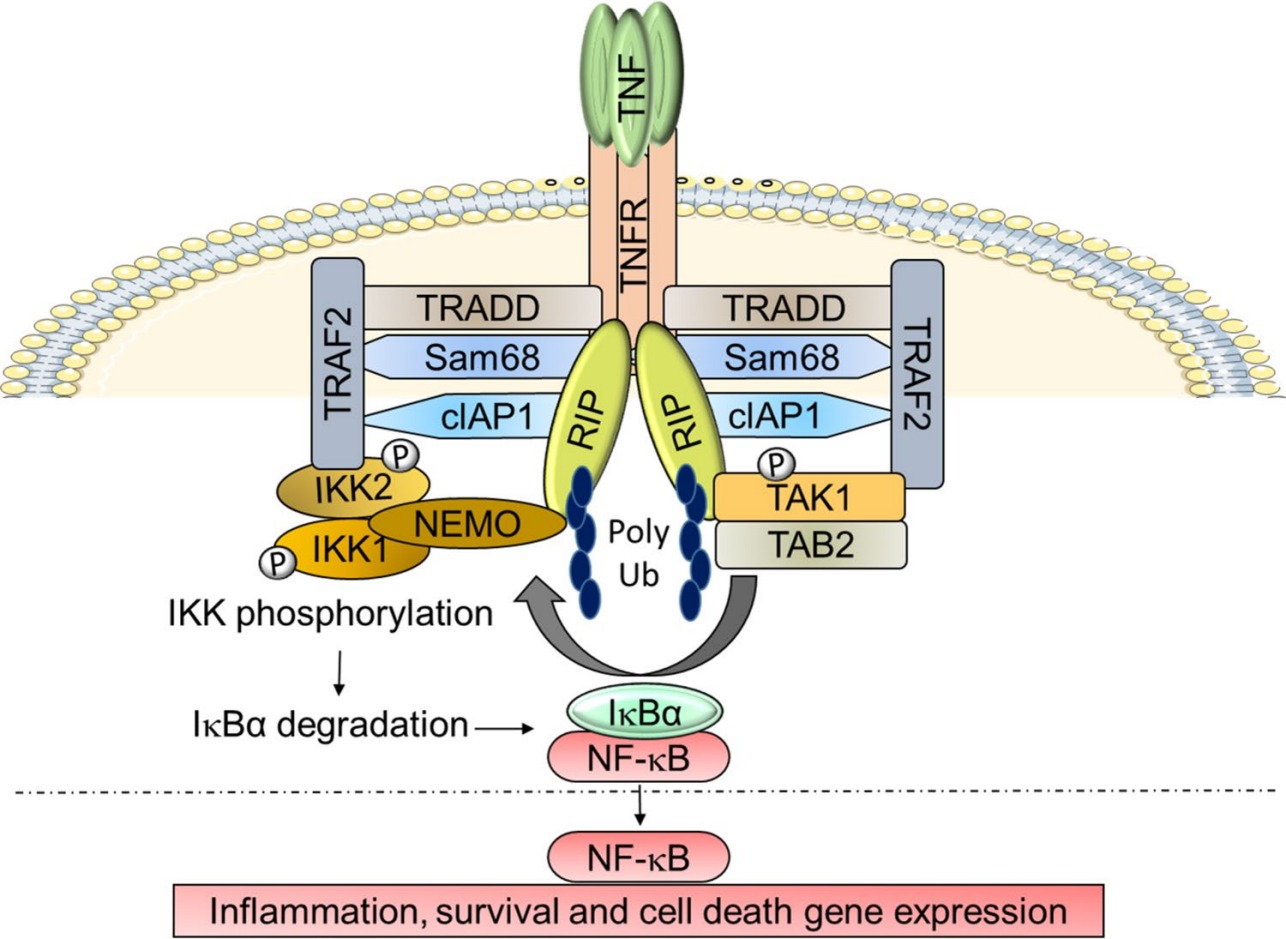
Schematic representation of the role of Sam68 in TNF-induced NF-κB signaling. TNF stimulation triggers the recruitment of indicated signaling proteins to the TNFR. Sam68 directly binds to TNFR, TRAF2, and RIP at the TNFR. Sam68 and ubiquitin chains on RIP aid in proper orientation of the TNFR complex. Proximity-induced TAK1 activation phosphorylates the IKK complex, which phosphorylates IκBα, leading to its ubiquitylation and degradation, liberating the bound NF-κB, that translocated to the nucleus. *P* phosphorylation, *Ub* ubiquitylation

TNFR1 is a ubiquitously expressed cell surface receptor associated with a variety of inflammatory and autoimmune pathologies [56]. TNF stimulation induces binding of Sam68 to the TNFR1 receptor [6], which then results in rapid activation of a complex containing TRADD, TNF receptor-associated factor 2 (TRAF2), cIAP1/2, and receptor-interacting protein kinase 1 (RIPK1) to instigate the activation of the signalosome complex containing IKKα, IKKβ and NEMO. Activated IKKβ then phosphorylates the inhibitory subunit of NF-κB, or IκB, leading to its proteolytic degradation and NF-κB activation (Fig. 5). With prolonged TNF stimulation, a second complex containing Sam68, RIPK1, FADD, and caspase 8 is formed to direct the signal toward an apoptotic cellular response [6]. Sam68, as an adaptor, is required upstream for the proper formation and activation of these NF-κB and apoptosis activating complexes. When Sam68 is removed or inhibited, TNFR1 demonstrates ablated inflammatory/death signaling responses (Fig. 5) [6, 20]. In vitro binding studies have shown that Sam68 directly binds to TNFR, TRAF2, and RIPK1, and preliminary deletion studies show that the absence of the KH domain region in Sam68 compromised the protein’s binding to the TNFR [6]. Because these bindings are likely not directly dependent upon the SH2/SH3-binding potential of Sam68, further biochemical characterization is necessary to decipher the exact binding domains of Sam68 that allows its adaptor role in the TNFR1 pathway. It is also possible that there is an accessory protein which links Sam68 to TNFR1. An example may be Grb2 with the SH2/SH3 domains, as it has previously been shown to partake in TNFR1 signaling [57]. Finally, the apoptotic function of Sam68 has been shown to be negatively regulated by SUMOylation at its N terminus [9]; however, how SUMOylation affects protein–protein interactions and adaptor function of Sam68 warrants detailed future studies.

### Role of Sam68 in TLR signaling

**Fig. 6:**
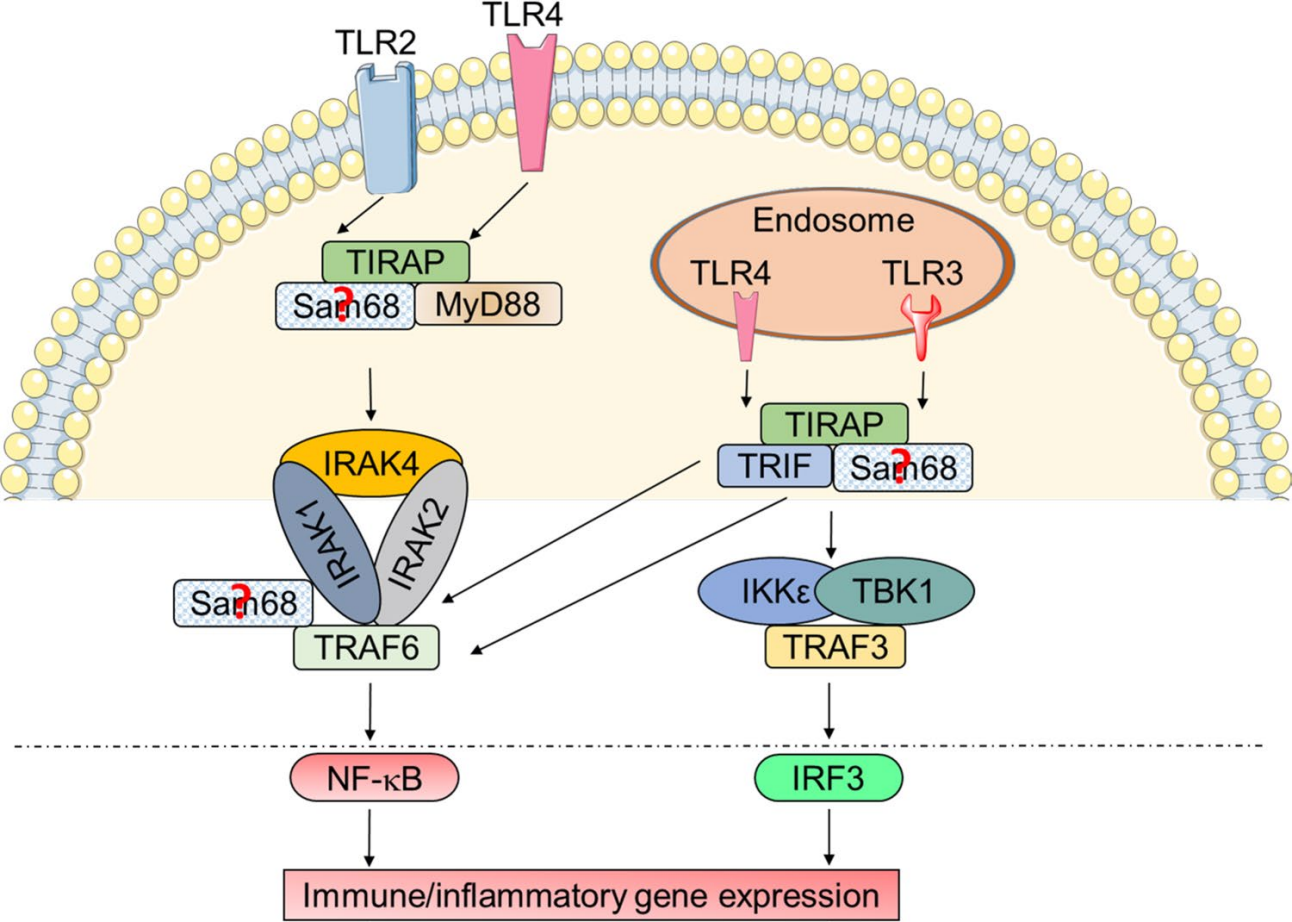
Schematic representation of role of Sam68 in TLR2, TLR3, and TLR4 signaling pathways. Sam68 plays an important role as an adaptor protein in TLR2- and TLR3-induced NF-κB activation and inflammatory gene induction and is partially required for TLR4 signaling. Potential binding complexes which may contain Sam68 are indicated by a question mark (?)

The TLR family is a major component of the innate immune system implicated in a variety of autoimmune and inflammatory diseases with both pro- and anti-inflammatory roles [58–61]. Sam68 has been shown to be required for the activation of two TLR2 pathways (TLR2/1 and TLR2/6) and TLR3 activation, and is partially required for TLR4 signaling (Fig. 6) [17]. During pathologic inflammation driven by TLR activation, Sam68 appears to serve as a crux in both MyD88 and TRIF adapted cascades downstream of the TLRs. Furthermore, it has been found that when TLR2 and TLR3 are activated in Sam68-deficient cells, a differential nuclear translocation of NF-κB subunits is observed; while c-Rel translocation was found dependent on Sam68, p65 translocation showed only partial Sam68 dependence. Sam68 was shown to be essential for the expression of selected genes following TLR2 activation, yet not TLR3 activation, in macrophages. This suggests a plausible non-redundant transcriptional role for c-Rel containing NF-κB dimers activated by TLR2 in a Sam68-dependent manner. It appears that in case of TLR3 activation, the absence of Sam68 and resulting loss of nuclear c-Rel may be partially compensated by p65 containing dimers (Fig. 6) [17]. While it has been demonstrated that both MyD88- and TRIF-dependent signaling require Sam68 for optimal NF-κB activation, the exact binding partners of Sam68 or protein-complex(es) containing Sam68 at the receptor level or in the cytoplasm in TLR signaling remain unknown. It would be interesting to learn the biochemical and mechanistic differences in signaling involving Sam68 at the endosomal level by comparatively studying TLR2 vs TLR3 signaling that signals through MyD88 and TRIF, respectively. In addition, how Sam68 functions in the two complexes associated with TLR4, one at the membrane and another at the endosome, also warrants future investigation (Fig. 6). Furthermore, much like what is observed in Leptin signaling, TLR signaling is known to readily induce TNF production [62], which would further contribute to a positive-pro-inflammatory feedback loop maintained by Sam68 adapting multiple overlapping pathways.

### Role of Sam68 in inflammatory diseases

**Fig. 7:**
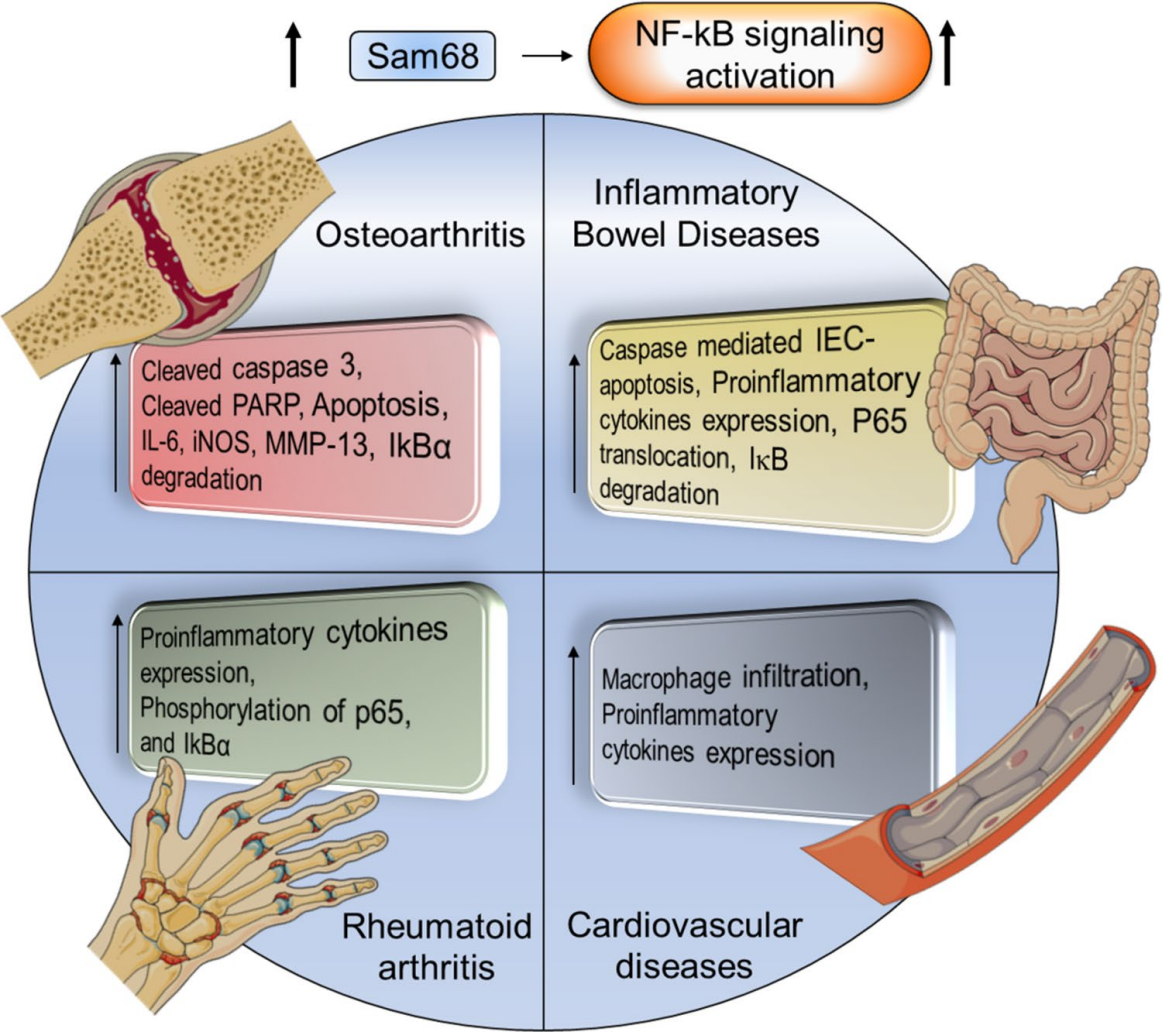
Role of Sam68 in various chronic inflammatory disorders. An increase in Sam68 levels or function is correlated with enhanced NF-κB-mediated inflammatory signaling in osteoarthritis, inflammatory bowel disease, cardiovascular diseases, and rheumatoid arthritis. Known inflammatory complications associated with these diseases are depicted

Sam68 functions as a scaffold in signal transduction pathways and transcription complexes through protein–protein interactions, controlling a variety of biological activities. Sam68 is required for both TNF and TLR-mediated NF-κB signaling [6, 17], and a number of studies have found it to be a key inflammatory driver in a variety of pathophysiological processes, including chronic auto-inflammatory disorders such as UC [19], arthritis [20], and arterial injury [21] (Fig. 7). Overall, Sam68 overexpression correlates with chronic pro-inflammatory NF-κB signaling and increased apoptosis [5, 19–21].

### Arthritis

Osteoarthritis (OA) is the most common musculoskeletal disorder and the leading cause of disability worldwide [63]. Although OA is characterized by progressive destruction of articular cartilage [64], it is also associated with a complex pathogenesis that affects all tissues within the joint [65]. It has been reported that chronic inflammation and apoptosis have close relationships with OA progression [66, 67]. Multiple evidence indicates the potential role of Sam68 in NF-κB activation and inflammation as well as in TNF-receptor mediated apoptosis [6, 17, 21]. Sam68 expression has been shown to be elevated in OA cartilage tissues and chondrocytes upon TNF stimulation, along with increased expression of apoptotic markers such as cleaved caspase-3 and cleaved PARP [20]. Sam68 deletion significantly reduced the expression of the apoptotic markers and NF-κB dependent catabolic markers such as interleukin-6 (IL-6), A disintegrin and metalloproteinase with thrombospondin motifs (ADAMTS)-5, inducible nitric oxide synthase (iNOS) and matrix metalloproteinase (MMP)-13, as well as hindered IκB degradation and nuclear translocation of NF-κB p65. These findings also align with the suggested adaptor role of Sam68 in the TNFR signaling complex leading to NF-κB activation and apoptosis induction [6], executing a critical role in the pathophysiology of OA [20].

Rheumatoid arthritis (RA) is another chronic, destructive form of arthritis which is characterized by polyarthritis, joint destruction, and functional impairment [68]. Complex networks of pro-inflammatory cytokines, chemokines, and growth factors play a fundamental role in its pathogenesis. However, given the considerable variation in clinical courses and therapeutic responses of RA patients, the precise pathophysiological pathways involved in the etiology and progression of RA is only partially understood [69]. NF-κB signaling pathway mediated inflammation plays a key role in the pathogenesis of RA [70]. The relevance of Sam68 in this pathway appears to rest in its ability to bind to the p65 subunit of NF-κB in the nucleus, promoting transcription of several NF-Κb-dependent inflammatory genes [16, 71]. However, it remains unknown as to how Sam68 modulates NF-κB-DNA interactions. It is possible that Sam68 alters the NF-κB dimer composition, modifies the affinity of the NF-κB-DNA interaction, or directly interacts with the NF-κB-binding regions on the promoter region to alter transcription. Sam68 expression was shown to be markedly elevated in RA patients' synovial tissue, where it co-localizes with THY1, a marker for fibroblast-like synoviocytes (FLS), indicating that overexpression of Sam68 contributes to pathology of synovial inflammation in RA [5]. In addition, Sam68 expression was increased in FLS in response to TNF stimulation in a time-dependent manner. Suppression of Sam68 by siRNA dramatically reduced the expression of TNF-induced cytokines and FLS proliferation, migration, and invasion, along with reduced phosphorylation of p65 and IκBα. Collectively, these data highlight Sam68's role in RA inflammation through NF-κB signaling pathway [5] and leave opportunity for mechanistic and translational studies focusing on Sam68 open for future investigations.

### Inflammatory bowel diseases (IBD)

IBD, which include UC and Crohn's disease (CD), are chronic mucosal inflammatory disorders of the intestine [72, 73]. The intestinal epithelial cells (IEC) regulate intestinal homeostasis by participating in coordinated responses to signals from the microbiota and local leukocyte populations [74, 75]. Compromised epithelial homeostasis associated with increased cytokine production, leukocyte infiltration, and IEC apoptosis are suggested factors of IBD pathogenesis; however, the molecular mechanisms involved remain poorly defined [76–78]. Therefore, identification of novel signaling mechanisms in IBD remains a challenge [19].

Previous works have identified critical roles of TNFR and TLR mediated pro-inflammatory signaling in IBD [58, 79]. Strategies targeting TNFR to control inflammation are among the frontline therapeutic strategies for IBD [80], but they often result in partial success and a high rate of relapse [81]. Targeting TLR pathway is another emerging potential therapeutic approach for treating IBD [58]. Sam68 has been reported as a critical mediator of both TNFR and TLR mediated signaling [6, 17]. Although Sam68 has shown to be necessary for TLR-induced inflammatory signaling, the precise, mechanistic role and the biochemistry of Sam68 in TLR signaling has not been studied in detail. In contrast, sufficient foundational knowledge exists on the mechanistic role of Sam68 in TNFR signaling [6]. Absence of Sam68 was shown to compromise TNF-induced inflammation in human colon cell lines and in DSS-induced experimental mouse IBD model [19, 82] and global deficiency of Sam68 was shown to protect mice from colon inflammation and development of experimental colitis [19]. Of note, both the levels of Sam68 and pro-inflammatory gene expression were reported to be significantly elevated in the colon biopsies of UC patients, suggesting a potential role of Sam68 in UC pathogenesis [82]. Whether elevation of Sam68 is a cause or consequence of UC is an interesting question that is worthy of investigation. In addition to its role in NF-κB activation and inflammatory signaling, Sam68 also binds to the TNF-induced apoptosome complex containing caspase 8, mediating programmed cell death [6]. IEC damage, recovery, and death are perpetuating events in UC and there has yet to be a comprehensive study on how Sam68 expression levels and functions regulate these processes. Studies on the possible roles of Sam68 in the mechanisms involved in UC showed that it is required for NF-κB activation promoting catabolic gene expression and caspase-mediated IEC apoptosis [82]. Sam68 deficiency in HT29 cells significantly reduced TNFα-induced Iκ-Bα degradation and attenuated p65 nuclear translocation, corroborating earlier studies and further demonstrating Sam68's critical function in IEC apoptosis and the UC pathogenesis through NF-κB-mediated inflammation [82]. Initial study of cell type specific function of Sam68 suggests that hematopoietic cell-specific Sam68 may not play a substantial role in the pathogenesis of acute experimental colitis in mice, alluding to Sam68’s function in the non-hematopoietic compartment as a regulator of intestinal inflammation [19]. Further studies using IEC-specific Sam68 knockout mouse models are necessary to delineate the epithelial role of Sam68 in IBD. Overall, given its function in TNF- and TLR-mediated inflammation, Sam68 may prove a potential therapeutic target for the treatment of UC and potentially IBD with fewer side effects than the global TNF inhibition currently recommended in the clinic for IBD.

### Cardiovascular diseases

The function of Sam68 in cardiovascular biology is not yet well understood. Sam68 has been shown to be involved in vascular inflammatory response to denudation injury, likely through its role in TNF/NF-κB signaling. Sam68 interacts with Filamin A (FLNA) to stabilize TRAF2 on the cytoskeleton, which in turn amplifies NF-κB signaling and encourages the inflammatory response in arterial damage [21]. Sam68 deletion was shown to reduce neointima hyperplasia, associated with a reduction in macrophage infiltration and downregulation of pro-inflammatory genes in damaged arteries. Moreover, bone marrow transplantation from Sam68-deficient mice to WT mice increased vascular remodeling, suggesting a potential proinflammatory role of Sam68 in hematopoietic cells in regulating vasculature [21]. The question as to whether the positive regulation of vascular remodeling by Sam68 deficient bone marrow cells demonstrates an inhibitory role of Sam68 in this process remains unanswered. Apart from cytokine and immune cell-mediated inflammation, Sam68 has also been shown to regulate angiotensin II (ANG II) receptor signaling in vascular smooth muscle cells (VSMC). ANG II stimulation increases Sam68 phosphorylation and its binding to PI3K and these processes are associated with altered VSMC growth and vascular changes in hypertension, which is one of the prominent risk factors in cardiovascular diseases [83]. The upstream kinases that phosphorylate Sam68, precise role of cytoplasmic or nuclear Sam68 as well as RNA-binding dependent and independent roles of Sam68 in regulating VSMC growth in hypertension, are all topics that remain to be explored.

### Sam68 as a potential therapeutic target

Sam68's emergence as a key STAR protein is the result of its apparent multifunctionality as an RNA-binding protein and signaling adaptor, mediating protein–protein interactions in multiple pathways in various cell types [84]. Several reports also indicate that Sam68 undergoes various posttranslational modifications which can alter its function in propagating signal transduction [6]. Sam68 is downstream of the TNFRs [6], hepatocyte growth factor (HGF)/Met receptor [85], leptin, insulin, and epidermal growth factor (EGF) receptors [86, 87]. Moreover, emerging evidence reveals the potential of Sam68 to control the pathological processes of ataxia, osteoporosis, cancer, obesity, and IBD [87].

Sam68 has also been documented as a Cyclic Adenosine Monophosphate-Response-Element-Binding protein (CREB)-Binding Protein (CBP) binding partner which can repress abrupted Wnt/β-catenin signaling. As a result, Sam68 has been identified as a selective target and mediator of Wnt/β-catenin regulation in human cancer stem cells [88]. Abnormalities in the WNT/β-catenin pathway have been implicated in the manifestation of many types of human diseases including cancer [88, 89]. Treatment of cells with the ICG and CWP families of small molecules (such as ICG-001 and CWP232291) mechanistically disrupts the interaction between CBP and β-catenin via the promotion of a Sam68/CBP complex, which in turn alters CBP/β-catenin-dependent transcription to promote apoptosis and differentiation [88]. Similarly, CWP232228, an analog of the inhibitor ICG-001, a compound related to CWP232291, has also been reported to induce the Sam68–CBP complex formation, impairing transcription of known Wnt pathway target genes [88]. A recent finding identified Sam68 as a primary target of reverse-turn peptidomimetic small molecules (i.e., YB-0158) like CWP232228. YB-0158 treatment leads to the disruption of Sam68–Src protein interactions which results in significant accumulation of Sam68 in the nucleus. This nuclear shuttling of Sam68 enhances the formation of Sam68–CBP complexes, thereby avoiding the CBP/β-catenin interaction altogether [90]. Sam68 has also been suggested to be a potential target of sunitinib malate, a widely used first‐line drug for metastatic renal cell carcinoma (RCC) [91]. Wu et al., have demonstrated that sunitinib treatment significantly inhibits the total expression and phosphorylation of Sam68 in RCC cell lines. As further evidence, sunitinib sensitivity in ccRCC (clear cell renal cell carcinoma) patients was found to be dependent on increased Sam68 expression levels in sunitinib-sensitive RCC tumor tissues when compared to sunitinib-resistant tissues [91].

### Conclusions and future directions

Current knowledge of Sam68’s role in regulating protein–protein interactions and signal transduction is limited, with most of its functions attributed to RNA binding. With no enzymatic activity and an abundance of spatially distributed functional domains such as proline-rich regions, multiple tyrosine phosphorylation sites and nuclear localization signal, Sam68 earns the apt title of an ubiquitous adaptor protein. The multiple arginine methylation sites of Sam68 may also regulate protein–protein interactions which will serve to further broaden the spectrum of potential binding partners of Sam68 [92]. One key area which warrants future investigation is the identification of discrete functional domains and/or posttranslational modifications of Sam68 that dictate its RNA-binding and protein-binding roles in a cell type and disease-specific manner. It is also important to investigate the unique and overlapping roles of Sam68 in immune and inflammatory signaling pathways, a necessity when designing specific therapeutic agents targeting distinct functions of Sam68 such as inflammation, cell proliferation, and cell death. Because Sam68 is an intracellular protein, strategies to develop therapeutic compounds targeting Sam68 that are cell permeable with adequate stability and bioavailability are essential. Thus, there exists ample need and large scope for further comprehensive studies on Sam68 which may lead to substantial new therapeutic opportunities targeting this STAR protein in chronic inflammatory diseases.

## Data Availability

This review does not contain any new original data. All data discussed here are cited in the references.
